# Serum high-sensitive C-reactive protein is a simple indicator for all-cause among individuals with MAFLD

**DOI:** 10.3389/fphys.2022.1012887

**Published:** 2022-10-20

**Authors:** Jiaofeng Huang, Mingfang Wang, Yinlian Wu, Rahul Kumar, Su Lin

**Affiliations:** ^1^ Department of Hepatology, Hepatology Research Institute, The First Affiliated Hospital, Fujian Medical University, Fuzhou, Fujian, China; ^2^ Fujian Clinical Research Center for Liver and Intestinal Diseases, Fuzhou, Fujian, China; ^3^ Department of Gastroenterology and Hepatology, Duke-NUS Academic Medical Centre, Changi General Hospital, Singapore, Singapore

**Keywords:** MAFLD, C-reactive protein, mortality, NHANES, NAFLD

## Abstract

High-sensitive C-reactive protein (hs-CRP) is one of the diagnostic components for metabolic (-dysfunction) associated fatty liver disease (MAFLD). This study aimed to explore the relationship between hs-CRP level and 25-year mortality in patients with MAFLD. The study data were from the Third National Health and Nutrition Examination Survey 1988–1994. All participants were followed up until December 2015 and the outcome of each participant was ascertained from National Death Index records. Cox proportional hazards models were used to estimate hazard ratios (HRs) and 95% confidence interval (CI) of all-cause mortality, cardiovascular-related mortality, and malignancy-related mortality. A total of 4,145 participants with MAFLD were included in final analysis. The median follow-up period was 22.3 years (interquartile range 16.9–24.2). There were 1,610 (38.8%) all-cause deaths. The leading cause of death was malignant neoplasms (365/1,610, 22.7%), followed by cardiovascular diseases (342/1,610, 21.2%). Of the 4,145 patients with MAFLD, 1,293 (31.2%) had an hs-CRP level greater than 0.5 mg/dl. Those with hs-CRP > 0.5 mg/dl were older, more likely to be female and had greater derangements of metabolic profiles than those with lower hs-CRP. The results of Cox regression analysis showed that hs-CRP ≥ 0.5 mg/dl was an independent risk factor for all-cause mortality (HR = 1.394, 95% CI 1.253–1.551), cardiovascular mortality (HR = 1.497, 95% CI 1.190–1.885) and malignant neoplasms related mortality (HR = 1.290, 95% CI 1.030–1.615) after adjusting for risk factors. This study confirms that hs-CRP is an independent predictive factor of poor prognosis in patients with MAFLD.

## Introduction

Metabolic (-dysfunction) associated fatty liver disease (MAFLD) is the leading cause of chronic liver disease, affecting approximately 25% of the world population (Younossi et al., 2016; Lin et al., 2021). The prevalence of MAFLD is expected to increase in the forthcoming decades (Mantovani et al., 2020; Paik et al., 2020). MAFLD may eventually lead to cirrhosis, liver cancer or end-stage liver disease (Younossi et al., 2019; Kanneganti and Singal, 2022). Moreover, MAFLD is also linked to extrahepatic diseases, such as cardiovascular disease, extra-hepatic cancers, and chronic kidney disease (Alharthi and Eslam, 2022; Tao et al., 2022). MAFLD has been demonstrated to be associated with increased all-cause mortality in both the United States (Kim et al., 2021) and China (Wang et al., 2022). As the outcomes of patients with MAFLD is not optimal, identification of high-risk patients is paramount for the management of MAFLD.

C-reactive protein (CRP) is an acute-phase protein synthesized by the liver. It will increase significantly in the inflammatory states, such as inflammation, infection or tissue injury. Meanwhile, it is frequently used as a marker for inflammation in routine clinical practice (Bruserud et al., 2020). It can also be seen in chronic diseases or in long-lasting inflammation and is regarded as a marker of systemic inflammation. CRP has been proposed for its potential to add a predictive value in several chronic disease (Liu et al., 2020; Chen et al., 2022). High-sensitive CRP (hs-CRP) permits the detection of lower level of CRP. Although hs-CRP is one of the components for the diagnosis of MAFLD, its prognostic role in MAFLD remains unknown. The aim of this study was to prospectively examine the role of hs-CRP in prediction of all-cause and cause-specific mortality in patients with MAFLD.

## Methods

### Study design and ethics

All analyses were based on the data from the Third National Health and Nutrition Examination Survey (NHANES III), which was a national survey study conducted by the National Center for Health Statistics (NCHS) between 1988 and 1994. The participants were all followed up until December 2015. The survival data had been uploaded online and was freely for researchers to use. All study procedures of NHANES III were approved by the Institutional Review Board of the NCHS, Centers for disease Control and Prevention. The study only utilized publicly available anonymous data; therefore, further ethics approval was not required. All dataset used in this study can be achieved on the NHANES website. (https://www.cdc.gov/nchs/nhanes/index.htm).

### Definition

MAFLD was diagnosed by the presence of both abdominal ultrasound confirmed hepatic steatosis and any of the following three conditions: overweight/obesity, type 2 diabetes, or evidence of metabolic dysregulation (Eslam et al., 2020).

### Demographic variables

The following demographic variables were obtained from the original database: age, gender, race, weight, height, history of hypertension and type 2 diabetes. Body mass index (BMI) was calculated as the weight/height^2^ ratio (Kg/m^2^).

### Laboratory examinations

In the NHANES III cohort, hs-CRP was measured using a high-sensitivity assay with latex-enhanced nephelometry. The levels of hs-CRP below 0.21 mg/dl were undetectable in the NHANES III. As the mean of hs-CRP in the MAFLD individuals was 0.54 mg/dl, for convenience we took the cutoff value of 0.5 mg/dl to group patients. Patients with MAFLD were divided into two groups according to cut-off values of 0.5 mg/dl of hs-CRP. Baseline laboratory tests assessed were platelet count, serum aspartate aminotransferases (AST), alanine transferases (ALT), fasting blood-glucose (FPG), fasting insulin, serum triglyceride, total cholesterol, high-density lipoprotein cholesterol (HDL-C), and glycosylated hemoglobin (HbA1c). Homeostasis model assessment insulin resistance (HOMA-IR) indexes were calculated by the following formula: HOMA-IR scores = FPG × fasting insulin/22.5. Detailed descriptions of the laboratory protocols are provided in the NHANES III laboratory procedures manual.

### Hepatic steatosis assessment

The ultrasound examinations were performed on adults aged 20–74 years who were examined in NHANES III. A detailed description of the protocol was provided in the procedure manual. (https://wwwn.cdc.gov/nchs/data/nhanes3/34a/HGUHS.htm). Categorized assessment of hepatic steatosis by ultrasound in the NHANES III was encompassed as none, mild and moderate to severe steatosis. Only mild to severe hepatic steatosis was regarded as evidence of hepatic steatosis.

### Non-invasive models for liver fibrosis

The AST to platelet ratio index (APRI), Fibrosis-4 score (FIB-4), NAFLD fibrosis score (NFS) and BARD score were used for noninvasive assessment of fibrosis (McPherson et al., 2010; Xiao et al., 2017; Siddiqui et al., 2019).

### Follow-up and mortality

Mortality status and cause-specific death was obtained from data linkage of NHANES dataset with the National Death Index. Follow-up time was calculated from interview date until date of death or end of study (31 December, 2015). The causes of death were confirmed based on ICD-9 codes in the year of 1998 and on International Classification of Diseases, 10th version (ICD- 10) codes for deaths occurring after 1998. To assist researchers with analyses that span the entire survey specific mortality period, in the original database, a UCOD_113 variable was created to recode all deaths into comparable ICD-10 based underlying cause of death groups (Anderson et al., 2001). In the present study, primary outcome was mortality from all causes, cardiovascular diseases (codes I00-I09, I11, I13, I20-I51, and I60-I69), and malignant neoplasms (C00-C97).

### Statistical analysis

Continuous variables are expressed as means ± standard variation. Categorical variables are expressed as percentages. The *t*-test (for normally distributed variables), Mann-Whitney *U*-test (for non-normally distributed variables) and Chi-squared test (for categorical variables) were used to investigate the differences between the groups. The Kaplan–Meier survival curves were plotted for all-cause, cardiovascular diseases, and malignant neoplasms mortality in individuals with MAFLD stratified according to serum hs-CRP levels. Hazard ratio (HR) and corresponding 95% confidence interval (CI) were estimated using multivariable Cox proportional hazards regression to examine the associations between serum hs-CRP level and mortalities. All tests were two-tailed and results with a *p* value less than 0.05 were considered statistically significant. All analysis was conducted using R 3.6.2 (https://www.r-project.org/).

## Results

### Baseline characteristics

**FIGURE 1:**
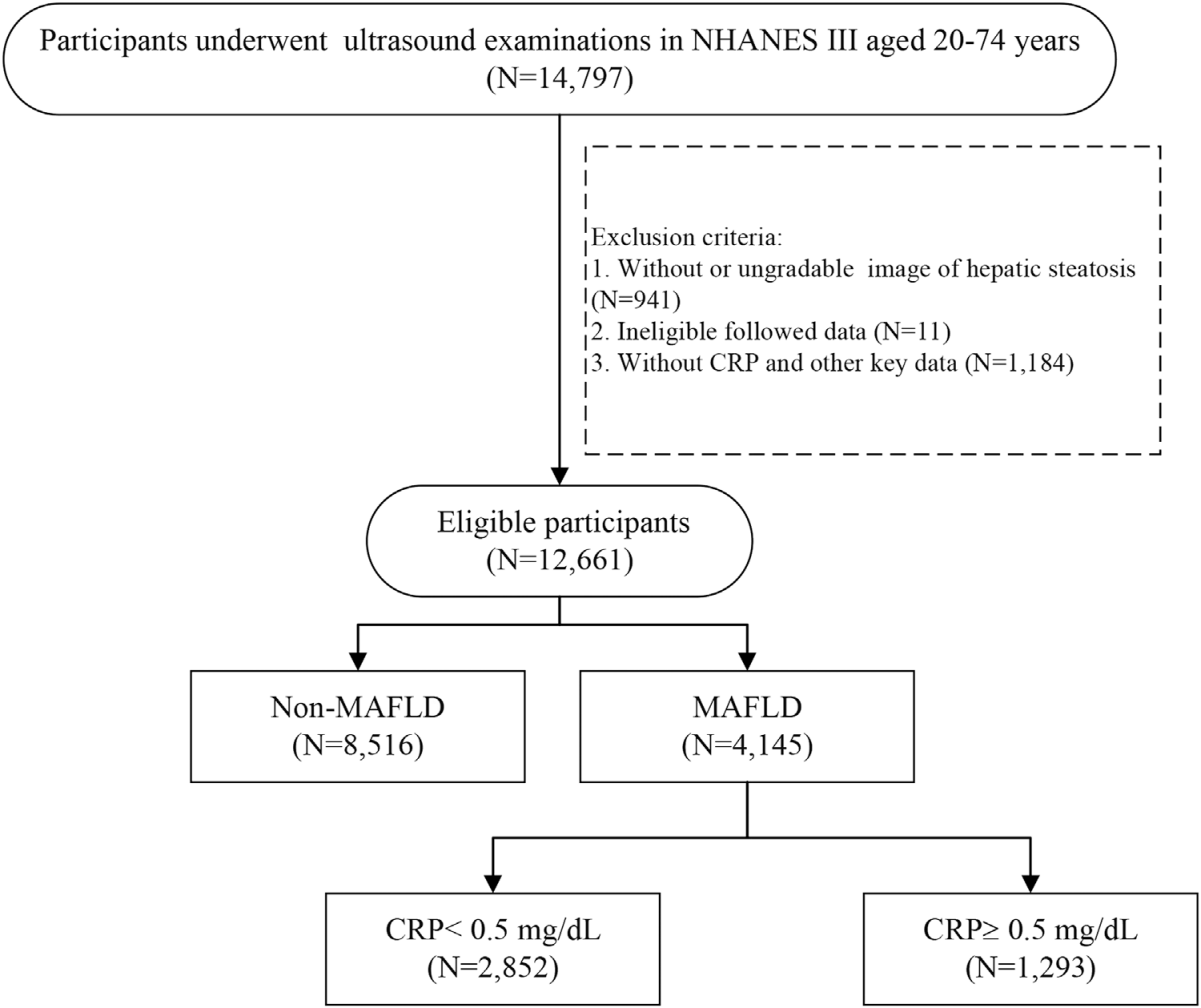
The flow chart of case selection.

From NHANES III, a total of 14,797 participants aged 20–74 years with assessment of abdominal ultrasonography initially fulfilled the predefined inclusion criteria. After excluding participants without eligible ultrasound data (*N* = 941), lost to follow-up (*N* = 11), and missing hs-CRP and other key data (*N* = 1,184), 12,661 participants were included for further analysis. According to the diagnosis of MAFLD, a total of 4,145 patients with MAFLD were included (Figure 1). The end of the follow-up date was 31 December 2015. The median follow-up period was 22.3 years (interquartile range 16.9–24.2). The proportion of male was 49.4% and the mean age was 48.0 ± 15.3 years. There were 1,610 (38.8%) all-cause deaths in the follow-up period. The leading cause of death in MAFLD participants were malignant neoplasms (365/1,610, 22.7%), followed by cardiovascular diseases (342/1,610, 21.2%).

### Comparison of the survival and death groups

The baseline characteristics of the study population between the survival and (all-cause) death groups were shown in the Table 1. The death group were older at baseline, had more male participants and higher prevalence of diabetes and hypertension. The metabolism-related profile, including FPG, HBA1c, HOMA-IR score, cholesterol, and triglyceride were higher in death group. As for non-invasive scores for liver fibrosis, including NFS, APRI, FIB-4 and BARD scores, were all higher in death participants (all with *p* value < 0.05). The patients in the death group were more likely to have an hs-CRP level ≥0.5 mg/dl than the survival group (38.3% vs. 26.7%, *p* < 0.001).

**TABLE 1:** Comparison between the survival and death groups.

Variables	Total	Survival	All-cause death	P
Case number	4,145	2,535	1,610	
Follow-up (years)	22.3 (16.9, 24.2)	23.6 (22.4, 25.1)	13.8 (8.4, 19.2)	<0.001
Gender, n (%)				<0.001
Male	2048 (49.4)	1,160 (45.8)	888 (55.2)	
Female	2097 (50.6)	1,375 (54.2)	722 (44.8)	
Age (years)	48.0 ± 15.3	41.1 ± 12.7	58.7 ± 12.7	<0.001
Race, n (%)				<0.001
Non-Hispanic white	1,451 (35.0)	761 (30.0)	690 (42.9)	
Non-Hispanic black	961 (23.2)	597 (23.6)	364 (22.6)	
Mexican-American	1,568 (37.8)	1,054 (41.6)	514 (31.9)	
Other	165 (4.0)	123 (4.9)	42 (2.6)	
Type 2 diabetes, n (%)	1,114 (26.9)	411 (16.2)	703 (43.7)	<0.001
Hypertension, n (%)	2,199 (53.1)	1,149 (45.3)	1,050 (65.2)	<0.001
Overdrinking, n (%)	338 (8.2)	193 (7.6)	145 (9.0)	0.110
BMI (kg/m^2^)	30.3 ± 6.4	30.2 ± 6.5	30.3 ± 6.3	0.693
platelet (‘10^9^/L)	278.2 ± 73.2	284.1 ± 71.5	268.9 ± 74.9	<0.001
FPG (mmol/L)	6.1 ± 2.7	5.6 ± 2	6.9 ± 3.4	<0.001
HbA1c (%)	5.8 ± 1.4	5.6 ± 1.1	6.3 ± 1.7	<0.001
HOMA-IR score	2.8 (1.8, 4.7)	2.6 (1.7, 4.1)	3.2 (2.0, 5.9)	<0.001
Triglyceride (mmol/L)	2.1 ± 1.6	2 ± 1.4	2.3 ± 1.8	<0.001
Cholesterol (mmol/L)	5.5 ± 1.2	5.3 ± 1.1	5.7 ± 1.2	<0.001
HDL-C (mmol/L)	1.2 ± 0.4	1.2 ± 0.4	1.2 ± 0.4	0.179
AST (U/L)	25.2 ± 19.8	25 ± 19.1	25.6 ± 21	0.414
ALT (U/L)	23.5 ± 21.4	25.1 ± 23.4	21 ± 17.5	<0.001
NFS scores	−1.6 ± 1.7	−2.1 ± 1.5	−0.7 ± 1.6	<0.001
APRI scores	0.3 ± 0.5	0.2 ± 0.3	0.3 ± 0.6	0.005
FIB4 scores	1.1 ± 1.4	0.8 ± 0.6	1.4 ± 2.0	<0.001
BARD scores	3 (2, 3)	3 (2, 3)	3 (2, 3)	<0.001
0	133 (3.2)	117 (4.6)	16 (1)	
1	367 (8.9)	295 (11.6)	72 (4.5)	
2	1,282 (30.9)	838 (33.1)	444 (27.6)	
3	1730 (41.7)	1,051 (41.5)	679 (42.2)	
4	633 (15.3)	234 (9.2)	399 (24.8)	
Hs-CRP group (mg/dl)				<0.001
<0.5	2,852 (68.8)	1858 (73.3)	994 (61.7)	
≥0.5	1,293 (31.2)	677 (26.7)	616 (38.3)	

BMI, body mass index; FPG, fasting plasma glucose; HbA1c, glycosylated hemoglobin; HOMA-IR, homeostasis model assessment insulin resistance; HDL-C, high-density lipoprotein cholesterol; ALT, alanine aminotransferase; AST, aspartate aminotransferase; APRI, AST to platelet ratio index; BARD, BMI-AST/ALT ratio and diabetes score; FIB-4, fibrosis-4 index; NFS, NAFLD fibrosis score.

### Comparison between higher and lower High-sensitive C-reactive protein groups

The characteristics of participants with MAFLD according to serum hs-CRP concentrations in NHANES III were shown in Table 2. Of the 4,145 patients with MAFLD, there were 1,293 (31.2%) patients with hs-CRP greater than 0.5 mg/dl. The group with higher hs-CRP level were older and female preponderant than the group with lower hs-CRP. As expected, higher serum hs-CRP coincided with a higher prevalence of diabetes, hypertension, and higher levels of BMI, platelet, FPG, HBA1c, and HOMA-IR scores. The lipid profile (including triglyceride and HDL-C) was not significantly different between the two groups (*p* > 0.05). The higher hs-CRP group had a higher level of NFS and BARD scores, while there was no statistical difference of APRI and FIB-4 indexes. The risk of all-cause mortality (47.6% vs. 34.9%, *p* < 0.001), cardiovascular related mortality (10.5% vs. 7.2%, *p* < 0.001) and malignant neoplasms related mortality (10.1% vs. 8.2%, *p* = 0.049) increased with hs-CRP level elevation (*p* < 0.05).

**TABLE 2:** Comparison between the two hs-CRP concentrations groups.

Variables	CRP mg/dL		*P*
	<0.5	≥0.5	
Case number	2,852	1,293	
Follow-up (years)	22.6 (18.9, 24.4)	21.9 (13.5, 23.6)	<0.001
Gender, n (%)			<0.001
Male	1,583 (55.5)	465 (36.0)	
Female	1,269 (44.5)	828 (64.0)	
Age (years)	46.9 ± 15.5	50.2 ± 14.8	<0.001
Race, n (%)			<0.001
Non-Hispanic white	1,028 (36)	423 (32.7)	
Non-Hispanic black	607 (21.3)	354 (27.4)	
Mexican-American	1,102 (38.6)	466 (36.0)	
Other	115 (4.0)	50 (3.9)	
Type 2 diabetes, n (%)	625 (21.9)	489 (37.8)	<0.001
Hypertension, n (%)	1,470 (51.5)	729 (56.4)	0.004
BMI (kg/m^2^)	29.0 ± 5.5	32.9 ± 7.4	<0.001
Overdrinking, n (%)	248 (8.7)	90 (7.0)	0.067
Platelet (‘10^9^/L)	272.3 ± 70.3	291.2 ± 77.8	<0.001
FPG (mmol/L)	5.8 ± 2.2	6.8 ± 3.5	<0.001
HbA1c (%)	5.7 ± 1.2	6.3 ± 1.8	<0.001
HOMA-IR score	2.5 (1.7, 4.1)	3.56 (2.2, 6.2)	<0.001
Triglyceride (mmol/L)	2.1 ± 1.6	2.1 ± 1.4	0.157
Cholesterol (mmol/L)	5.4 ± 1.1	5.5 ± 1.2	0.015
HDL-C (mmol/L)	1.2 ± 0.4	1.2 ± 0.4	0.096
AST (U/L)	25.4 ± 19.4	24.9 ± 20.9	0.479
ALT (U/L)	24.1 ± 20.9	22.1 ± 22.4	0.007
NFS scores	−1.8 ± 1.6	−1.2 ± 1.7	<0.001
APRI scores	0.3 ± 0.4	0.3 ± 0.6	0.910
FIB4 scores	1.0 ± 0.9	1.1 ± 2.1	0.151
BARD scores			<0.001
0	118 (4.1)	15 (1.2)	
1	279 (9.8)	88 (6.8)	
2	1,032 (36.2)	250 (19.3)	
3	1,116 (39.1)	614 (47.5)	
4	307 (10.8)	326 (25.2)	
All-cause mortality, n (%)	994 (34.9)	616 (47.6)	<0.001
Cause-specific mortality			
Cardiovascular diseases, n (%)	206 (7.2)	136 (10.5)	<0.001
Malignant neoplasms, n (%)	234 (8.2)	131 (10.1)	0.049

BMI, body mass index; FPG, fasting plasma glucose; HbA1c, glycosylated hemoglobin; HOMA-IR, homeostasis model assessment insulin resistance; HDL-C, high-density lipoprotein cholesterol; ALT, alanine aminotransferase; AST, aspartate aminotransferase; APRI, AST to platelet ratio index; BARD, BMI-AST/ALT ratio and diabetes score; FIB-4, fibrosis-4 index; NFS, NAFLD fibrosis score.

### Kaplan-Meier survival analysis

**FIGURE 2:**
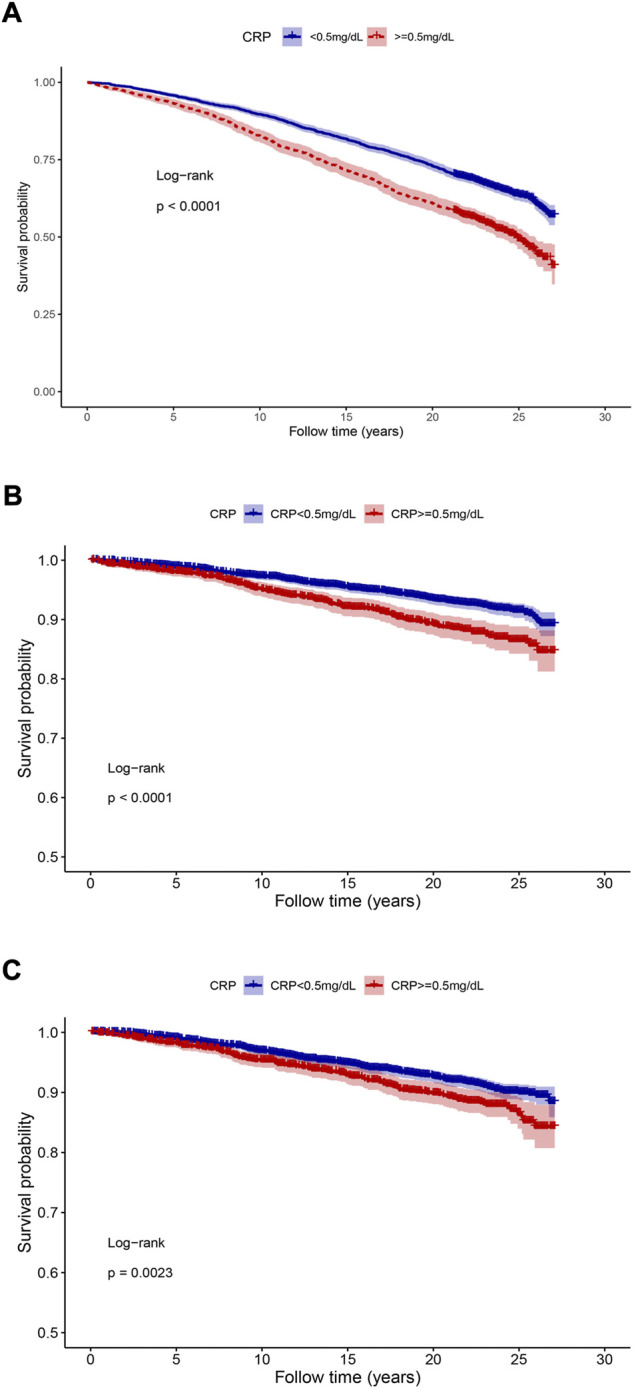
The Kaplan-Meier analysis between different hs-CRP concentrations and mortality. **(A)** All-cause mortality; **(B)** cardiovascular diseases related mortality; **(C)** malignant neoplasms related mortality. The *p-*values were calculated by the log-rank test.

The survival probability among the two hs-CRP level groups were visualized by Kaplan-Meier survival curve (Figure 2). The results showed that high hs-CRP level was associated with worse survival (Log-rank test, *p* < 0.05).

### Cox regression analysis

**FIGURE 3:**
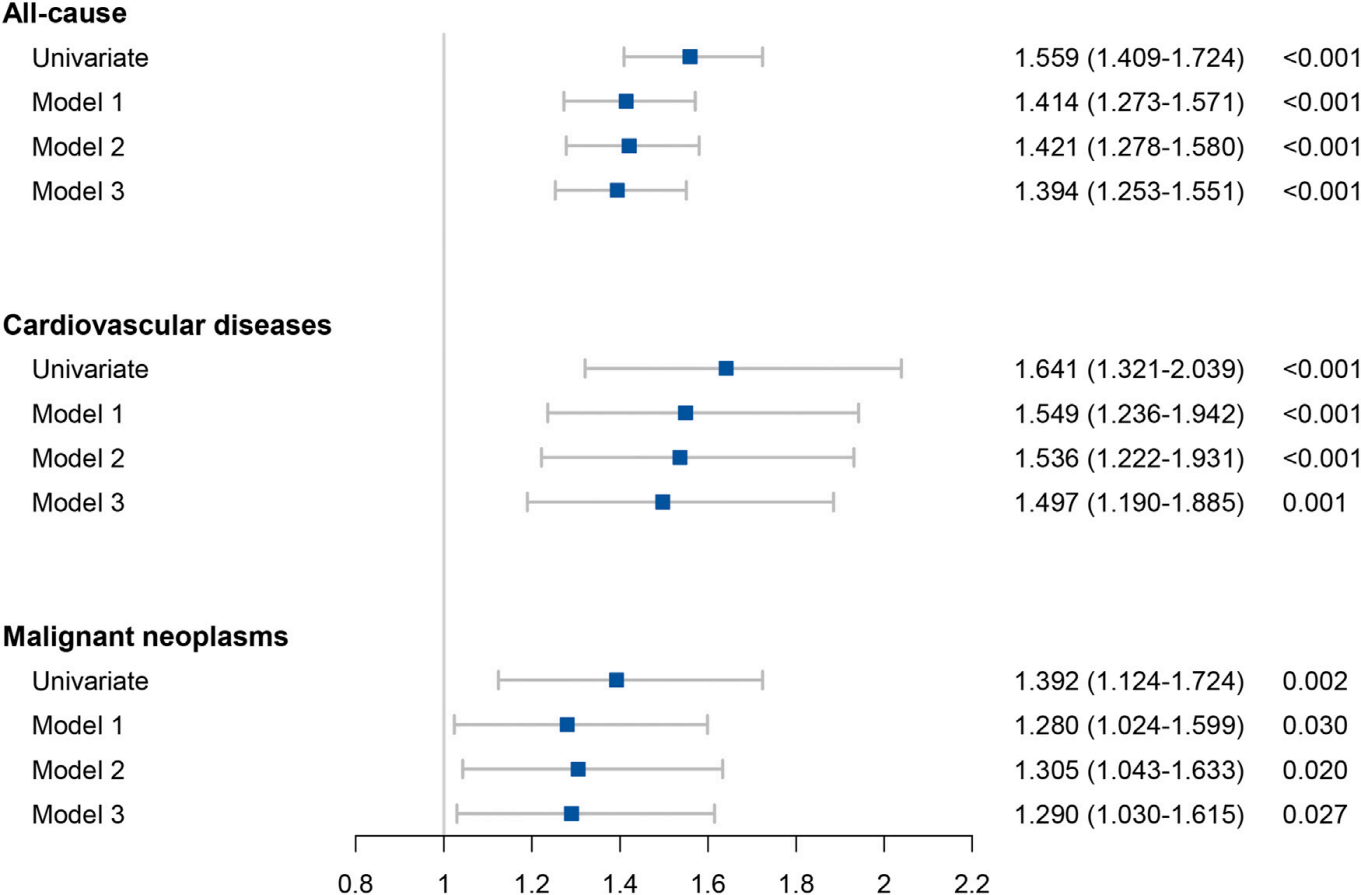
Forest plots of the results of all-cause mortality and cause specific mortality in Cox analyses.

A univariate and multivariate Cox regression analysis of the collected data was performed. Cox univariate analysis was performed on all variables and those with a *p*-value less than 0.05 in the univariate analysis were included in the multivariate Cox regression model. Model 1 was adjusted for gender, age (age≥40 years), race, diabetes and hypertension. Model 2 was adjusted for gender, age (age≥40 years), race, diabetes, hypertension, and continuous variable (including platelet, FPG, triglyceride and ALT). Model 3 was adjusted for Model 2 and FIB-4 scores. The results were showed in Table 3 and Figure 3. In all the three models, hs-CRP over than 0.5 mg/dl was an independent risk factors for all-cause, cardiovascular related mortality and malignancy related mortality. The results of Cox regression analysis showed that hs-CRP ≥ 0.5 mg/dl was an independent risk factor for all-cause mortality (HR = 1.394, 95% CI 1.253–1.551), cardiovascular related mortality (HR = 1.497, 95% CI 1.190–1.885) and malignant neoplasms related mortality (HR = 1.290, 95% CI 1.030–1.615).

**TABLE 3:** The hazard risk of hs-CRP (≥0.5 mg/dl) for mortality in patients with MAFLD.

Death	HR (95% CI)	P
All-cause mortality		
Univariate	1.559 (1.409–1.724)	<0.001
Model 1	1.414 (1.273–1.571)	<0.001
Model 2	1.421 (1.278–1.580)	<0.001
Model 3	1.394 (1.253–1.551)	<0.001
Cardiovascular mortality		
Univariate	1.641 (1.321–2.039)	<0.001
Model 1	1.549 (1.236–1.942)	<0.001
Model 2	1.536 (1.222–1.931)	<0.001
Model 3	1.497 (1.190–1.885)	0.001
Cancer mortality		
Univariate	1.392 (1.124–1.724)	0.002
Model 1	1.280 (1.024–1.599)	0.030
Model 2	1.305 (1.043–1.633)	0.020
Model 3	1.290 (1.030–1.615)	0.027

Model 1 adjusted for gender, age (age≥40 years), race, diabetes and hypertension.

Model 2 adjusted for gender, age (age≥40 years), race, diabetes, hypertension, and continuous variable (including Platelet, FPG, triglyceride and ALT).

Model 3 adjusted for gender, age (age≥40 years), race, diabetes, hypertension, continuous variable (including Platelet, FPG, triglyceride and ALT) and FIB-4 scores.

## Discussion

The predictive role of hs-CRP in MAFLD has not been fully investigated. In this nationally representative cohort study, we explored the role of hs-CRP in all-cause and cause-specific mortality in MAFLD. The results revealed that hs-CRP, one of the diagnostic components of MAFLD, could additionally serve as a biomarker for long-term survival for MAFLD.

In this study, hs-CRP level higher than 0.5 mg/dl was significantly associated with a higher risk of all-cause, cardiovascular and malignancy associated mortality in MAFLD. This association was independent of age, gender, race, medical history, metabolic profiles, and liver fibrosis scores. The findings of this study provide a simple biomarker to evaluate the risk of mortality of MAFLD.

We found that at the level of 0.5 mg/dl, hs-CRP could effectively distinguish MAFLD with poor prognosis. As shown in previous study, CRP higher than 1 mg/dl was regarded an indicator of acute inflammation (Ritchie et al., 1999). Hs-CRP further permits the detection of lower CRP. Increasing studies in the literature have indicated that elevated CRP concentration, even at a very low level, is strongly associated with the poor prognosis in many chronic diseases. Quan, et al. found even slightly elevated CRP (>0.22 mg/dl) was associated with 10-year mortality in Chinese patients with hyperglycemia (Qian et al., 2021). The same conclusion has been confirmed by several meta-analysis and cohort studies (Li et al., 2017; Kurl et al., 2021). We found similar result in this nationally representative cohort, which further emphasizes the usage of hs-CRP in predicting the prognosis of MAFLD.

One of the questions is whether the hs-CRP is only a mediator or an independent risk factor for the prognosis of MAFLD. CRP had been found to have complex interactions between several risk factors in elderly individuals, including smoking, diabetes, hypertension, BMI, and lipid metabolism (Nadrowski et al., 2016; Bruserud et al., 2022). All these factors are also associated with MAFLD. To clarify the independent role of hs-CRP in MAFLD, we adjusted potential confounders, including age, sex, race, metabolic profile, liver fibrosis stages and medical history. The associations between hs-CRP and mortality were not significantly attenuated after further adjusting for different confounders, suggesting hs-CRP was an independent predictive factor for the prognosis of MAFLD.

There are many possible explanations for the prognostic role of the elevated hs-CRP levels on risk of mortality in MAFLD. Low-grade inflammation is linked to the initiation and progression of MAFLD (Power Guerra et al., 2020; Francque et al., 2021; Zhang et al., 2022). Additionally, hs-CRP is a marker of senescence and aging, which is characterized by the accumulation of senescent cells that release proinflammatory cytokines, chemokines and other mediators that lead to inflammatory microenvironments in many tissues and organs (Bruserud et al., 2022). Moreover, CRP level is correlated to the endothelial dysfunction and may reflect the development of atherosclerosis (Della Corte et al., 2016; Maio et al., 2021). These evidences can explain the correlation between CRP and risk of cardiovascular and malignancy related morbidity and mortality.

Although there are some distinct advantages of our research like nationally representative data from the NHANES III, which allowed us to generalize our findings in a broader population and that we have adjusted for multitude of potential confounding factors, including age, gender, race, concomitant diseases, laboratory tests, and noninvasive fibrosis scores and an average follow-up period longer than 20 years, our study should be interpreted in light of some potential limitations. First, the identification of MAFLD was based on the steatosis discovered on liver ultrasonography rather than biopsy. However, liver biopsy is unethical for epidemiology studies of asymptomatic individuals. Ultrasonography is presently the most commonly used technique with high specificity to diagnose hepatic steatosis in clinical practice (Dasarathy et al., 2009). Second, due to the observational nature of the study, causation cannot be determined. Future *in vitro* and *in vivo* experiments should aim to elucidate the molecular mechanisms by which hs-CRP is associated with the prognosis of patients with MAFLD. Last, although the study population had been followed up for more than two decades, we had to admit that it was still a retrospective study. There were some cases who lost to follow up. Moreover, the detail of death cause especially the liver-related outcomes was not obtainable.

In conclusion, based on the NHANES III data, hs-CRP ≥0.5 mg/dl is an independent predictive factor for all-cause, cardiovascular and malignancy related mortality in MAFLD. Measurement of hs-CRP may provide accurate prediction of long-term outcomes of MAFLD.

## Data Availability

The original contributions presented in the study are included in the article/Supplementary Material, further inquiries can be directed to the corresponding author.

## Abbreviations

MAFLD, metabolic-associated fatty liver disease; hs-CRP, high-sensitive C-reactive protein; NHANES III, the Third National Health and Nutrition Examination Survey; BMI, body mass index; FPG, fasting plasma glucose; ALT, alanine aminotransferase; AST, aspartate aminotransferase; HbA1c, glycosylated hemoglobin; HOMA-IR, Homeostasis model assessment insulin resistance; HDL-C, high-density lipoprotein cholesterol; APRI, AST to platelet ratio index; BARD, BMI-AST/ALT ratio and diabetes score; FIB-4, fibrosis-4 index; NFS, NAFLD fibrosis score; HR, hazard ratio.
